# Diagnosing delirium in elderly Thai patients: Utilization of the CAM algorithm

**DOI:** 10.1186/1471-2296-12-65

**Published:** 2011-07-01

**Authors:** Nahathai Wongpakaran, Tinakon Wongpakaran, Putipong Bookamana, Manee Pinyopornpanish, Benchalak Maneeton, Peerasak Lerttrakarnnon, Kasem Uttawichai, Surin Jiraniramai

**Affiliations:** 1 Department of Psychiatry, Faculty of Medicine, Chiang Mai University, Chiang Mai, Thailand; 2 Department of Statistics, Faculty of Sciences, Chiang Mai University, Chiang Mai, Thailand; 3 Department of Family Medicine, Faculty of Medicine, Chiang Mai University, Chiang Mai, Thailand

## Abstract

**Background:**

Delirium is a common illness among elderly hospitalized patients. However, under-recognition of the condition by non-psychiatrically trained personnel is prevalent. This study investigated the performance of family physicians when detecting delirum in elderly hospitalized Thai patients using the Thai version of the Confusion Assessment Method (CAM) algorithm.

**Methods:**

A Thai version of the CAM algorithm was developed, and three experienced Thai family physicians were trained in its use. The diagnosis of delirium was also carried out by four fully qualified psychiatrists using DSM-IV TR criteria, which can be considered the gold standard. Sixty-six elderly patients were assessed with MMSE Thai 2002, in order to evaluate whether they had dementia upon admission. Within three days of admission, each patient was interviewed separately by a psychiatrist using DSM-IV TR, and a family physician using the Thai version of the CAM algorithm, with both sets of interviewers diagnosing for delirium.

**Results:**

The CAM algorithm tool, as used by family physicians, demonstrated a sensitivity of 91.9% and a specificity of 100.0%, with a PPV of 100.0% and an NPV of 90.6%. Interrater agreement between the family physicians and the psychiatrists was good (Cohen's Kappa = 0.91, p < 0.0001). The mean of the time the family physicians spent using CAM algorithm was significantly briefer than that of the psychiatrists using DSM-IV TR.

**Conclusions:**

Family physicians performed well when diagnosing delirium in elderly hospitalized Thai patients using the Thai version of the CAM algorithm, showing that this measurement tool is suitable for use by non-psychiatrically trained personnel, being short, quick, and easy to administer. However, proper training on use of the algorithm is required.

## Background

Delirium is a syndrome characterized by abrupt changes in attention and cognition [1], and is one of the most common and important conditions among elderly hospitalized patients. The prevalence of delirium has been reported to be between 22% to 60% among elderly patients, although the exact incidence has not been well established[1,2]. The elderly are at risk of suffering from delirium due to many factors (including their age, having multi-system illness, comorbidity, using many kinds of medication at the same time, and as a result of surgery). Infection, cardiovascular disease, metabolic disturbance and the use of substances or medication, as well as withdrawal from these substances, are examples of the causes of delirium [3]. Delirium is associated with poor treatment outcomes [4-10], such as morbidity, longer hospital stays, functional decline and mortality.

Many factors affect the prevalence of delirium in the elderly. For instance, diagnostic terms regarding delirium related conditions vary, and include acute confusional state, acute brain syndrome, alteration of consciousness, acute encephalopathy and hepatic encephalopathy (if the delirium is due to poor hepatic function), and delirium features themselves also vary; some patients may be lethargic (hypoalert or hypoactive delirium - often confused with depression) while others may be agitated (hyperalert or hyperactive delirium). Hyperalert delirium symptoms are normally recognized more by intake physicians or bedside nurses, while the less obvious symptoms of hypoalert delirium may often escape the clinician's attention. The fluctuation of delirium symptoms can be difficult to detect, especially if the examiner assesses the patients at a moment when they are free of such symptoms. As a result, awareness among the care team is crucial. It has been reported that between 33 and 64% of delirium patients can be diagnosed by general practitioners [11-15]; though even among attending physicians, the underdiagnosis of delirium in elderly hospitalized patients is quite common (68%), leading to delays in treatment[16]. Therefore, it is important to develop a tool that can be used to help recognize delirium among patients in this age group, one that is easy to use.

Among the instruments used for detecting delirium, the Confusion Assessment Method (CAM) [1] algorithm is a widely used and highly accurate delirium diagnostic tool [17], one which includes an instrument and a diagnostic algorithm for the identification of delirium. It was developed based on the DSM III-R criteria for use by non-psychiatrically trained staff [18]. The CAM instrument assesses the presence, severity and fluctuation of nine features of delirium, and the algorithm is based on four of these main features, which are: 1) the acute onset of mental status changes, 2) flutuating inattention episodes, 3) disorganized thinking, and 4) an alteration of consciousness. The diagnosis requires features 1 and 2, plus 3 or 4. The reported sensitivity of this tool ranges from 94 to 100% and its specificity from 90 to 95% [1], with high inter-rater reliability. It takes approximately ten to fifteen minutes to complete.

In addition, CAM has been translated into ten different languages[17], among them French[19], German[20] and Protuguese[21]. These translated versions have shown good validity in terms of detecting delirium across different populations. The authors thus view this tool as useful for diagnosing delirium in elderly Thai patients.

The purpose of the study is to investigate how well family physicians can detect delirium using the CAM algorithm, as compared to diagnosis by fully trained psychiatrists - which must be considered the gold standard.

## Methods

This study was approved by an independent IRB at the Faculty of Medicine, Chiang Mai University in Thailand. All the authors are certified in ICH-GCP training.

### CAM translation

Translation of the CAM followed translation and cultural adaptation. First, NW translated the CAM into Thai, after which, a bilingual translator who had never been exposed to the original CAM did a backward translation. Then, both translators reached a consensus after comparing and adapting the test so that its meaning was fully congruent with Thai culture.

### CAM training and inter-rater reliability

Assessing delirium using CAM requires training, and it should only be used with formal cognitive tests [17]. To ensure rater reliability with the Thai version of CAM, monthly training sessions were set up for the participating family physicians. The training sessions included a summary of delirium and a detailed explanation of the Thai version of CAM, including various cognitive tests (such as MMSE Thai 2002 and a digit span test). Inter-rater reliability was ensured by asking the participating physicians to watch videos of ten delirious patients randomly selected from various wards. Three psychiatrist and family physician pairings were chosen at the beginning of the study, remaining in this pairing until the end, including during the training process. During the training, the authors focused only on the assessment of CAM specific items and on scoring instructions, rather than the expertise of the doctors involved. Thus, the training was based on how to use CAM and how to score each CAM item according to each patient's clinical manifestation. There was no clinical training regarding delirium given as part of the exercise. Disagreements regarding delirium diagnoses occurred between the pairs, especially at the beginning of the training (but not when diagnosing non-delirious patients). Most of these disagreements could be attributed to the family physicians' inability to detect poor attention (CAM item 2A) and disorganized thoughts (CAM item 3). The training, which included providing feedback, was repeated until, in the last ten random cases (five delirious; five non-delirious), each pair reached a 100% agreement on the diagnosis.

### Participants and procedure

This study was a prospective validation study in 66 patients aged over 60 years of age newly admitted to a 2000-bed university-affiliated public hospital in Chiang Mai, Thailand. Over a five month period in 2009, a research nurse was responsible for screening and enrolling patients during the first 24 hours of their admission at the hospital. The nurse was given a list of each day's newly enrolled patients (with no diagnotic information included) and then randomly selected a small number of names each day from the list, for selection. After investigation, if the patients chosen turned out to have a previously diagnosed delirium condition, they were then excluded from taking part in the study. We also excluded patients admitted to the ICU with GCS < 8, or with significant hearing or visual impairments that would interfere with the testing process. For all patients chosen to take part, written informed consent was first received from the patient or their closest relative before proceeding.

The non-delirium experts used in this study were staff family physicians from the Department of Family Medicine at the Faculty of Medicine, Chiang Mai University, who spend most of their time carrying out general practitioner's work. On a day to day basis, they see patients within the setting of the primary care unit (PCU) at the hospital, under the jurisdiction of the Faculty of Medicine, as in Thailand, these PCUs are where residency training in Family Medicine takes place. On occasion, these physicians carry out outreach work within the community as part of an outreach community team. We invited these physicians to participate, as they represent a broad group of non-delirium experts, though they do play a role teaching residents how to detect delirium in the community.

MMSE Thai 2002 [22] was performed by a research nurse to record the participants' intitial cognitive functioning, and subsequently each patient was assessed twice at their bedside within 72 hours of admission by two assessors: a psychiatrist and a family physician. In order to avoid bias due to fluctuations in altered consciousness, both examiners randomly assessed each patient within 30 minutes of each other. Furthermore, the family physicians and psychiatrists used in this research had had no prior experience taking care of the study patients on a day to day basis, and since the participants were randomly selected by a research assistant, the doctors were given no prior notice or information regarding the patients' conditions, whether they were delirious or otherwise. All the psychiatrists used in this study had at least ten years of experience assessing psychiatric patients with DSMs, and the diagnosis of delirium by psychiatrists can be regarded as the gold standard. The family physicians used the Thai version of the CAM algorithm to evaluate the presence or not of delirium, and at the end of each interview, the examiners independently recorded their diagnoses. All the interviews were videotaped for both training and post-analysis purposes.

### Statistical analysis

Cohen's Kappa was used to calculate the extent of agreement between the family physicians and the psychiatrists, in terms of their diagnoses. The sensitivity and specificity of the Thai version of the CAM algorithm, as used by the family physicians, were calculated and compared to the diagnoses given by the psychiatrists using DSM-IV TR. Demographic variables and descriptive statistics (i.e. percentages) were reported, and a non-parametric statistical analysis was applied to data without a normal distribution, and in addition, the positive predictive value (PPV) and negative predictive value (NPV) were both determined. The Statistical Package for the Social Sciences (SPSS 17.0 for Windows) was used.

## Results

### Demographic data

Seventy-eight elderly hospitalized patients were originally enrolled for this study, of whom ten were excluded initially due to us not receiving informed consent from the relatives, and two were excluded because the interview was not completed. In total, 66 elderly completed the study, and the demographic data for these patients is shown in Table 1.

**Table 1:** The patients' demographics

Items	Values
Mean Age (years)	74.53 ± 8.07*
Min age (years)	60
Max age (years)	93
Number of males (%)	39 (59.1)
Mode of formal education (years)	4
Min education (years)	0
Max education (years)	16
Mean of MMSE (out of 30)	
% dementia by MMSE-Thai 2002	34(25.6)†
Comorbidity, n (%)	
Respiratory	2(3.2)
Cardio-respiratory	7(11.1)
Surgery	11(17.5)
Infection	6(9.5)
Metabolic	5(7.9)
Electrolyte imbalance	4(6.3)
Intracranial causes	6(9.5)
Blood dyscrasia	1(1.6)
Others	3(4.8)
More than one diagnosis	18(28.6)

N = 66, *There was a significant difference between the delirium and non-delirium group using both CAM algorithm and the DSM-IV TR (t = 3.0 vs. t = 2.3, p < 0.05), † Mann-Whitney U statistics indicated that there was a significant difference between the delirium and non-delirium groups using both the CAM algorithm and DSM-IV TR (p < 0.05)

### Sensitivity and specificity of the CAM algorithm used by family physicians

As can be seen in Table 2, the sensitivity of the CAM algorithm was found to be 91.9% while the specificity was 100.0%, when compared to the assessments carried out by psychiatrists using the DSM-IV criteria. The family physicians did not over-identify delirium. The positive predictive value of CAM was thus 100.0% and the negative predictive value was 90.6%. In terms of agreement between the raters, it was found that Cohen's Kappa yielded 0.91, p < 0.0001.

**Table 2:** Number of elderly patients with delirium

By CAM algorithm\By DSM-IV TR	Delirium	No delirium
Positive	34	0
Negative	3	29

Total	37	29

### Time spent by family physicians using the CAM algorithm

The amount of time the family physicians spent using the CAM algorithm was slightly but significantly different from the time the psychiatrists spent assessing the patients, with a Mean ± S.D. (min, max) = 7.77 ± 3.74 (2-20) for the physicians using CAM, and 8.86 ± 2.85 (3-15) for the psychiatrists' assessment interviews (p = 0.021, n = 66). Moreover, it was found that the level of agreement between the family physicians and the psychiatrists regarding delirium in dementia patients, as indicated by MMSE, was higher for the 'non-dementia' group than for the 'dementia' group (Cohen's K = 1.00, p < 0.0001, n = 16, and 0.86, p < 0.0001, n = 50, respectively).

## Discussion

The rate of delirium among the patients in this study was high (56.1%), and the majority of these cases (46.9%) were admitted to internal medicine units. Nearly one-third (28.6%) of the study patients had suffered from multiple physical diagnosis. We found that the patients with delirium had a significantly higher rate of dementia than those without delirium, and in addition, we found that the more advanced the age of the patients, the more delirium was found (a mean of 76-77 years in delirium patients vs. 71-72 years in non-delirium patients).

The results therefore demonstrated a satisfactory agreement between the diagnosis of delirium in elderly hospitalized patients by family physicians who used the CAM algorithm, and the psychiatrists' diagnoses, regardless of the dementia state (Cohen Kappa = 0.86-1.00). Two cases of discordance occurred when assessing the dementia group (case 1 achieved an MMSE score of 6/30, whereas the other yielded a score of 5/30). Both these cases were diagnosed with "delirium due to multiple etiologies" and positively identified as delirious only by the psychiatrists (meaning a false negative was produced by the CAM algorithm). Since delirium is a serious condition, a false negative diagnosis can be considered a worse result than a false positive. CAM3 (disorganized thought) seemed the most difficult item for the family physicians in terms of differentiating between delirium and non-delirium patients. This is considered serious, because it could result in the misdiagnosis of patients with an absence of item 4 (an alteration of consciousness). Therefore, in our view, more effort should be made during the training process to prevent any further false negative detection. In addition, after reviewing the videotape of the two cases, we found that the misdiagnosis originated from the fact that neither family physician used the proper cognitive assessment materials, as provided in the study. Such a common mistake has been previously reported [2] and may be preventable, especially when MMSE is used.

Looking at the assessment times, both groups of physicians averaged less than ten minutes to make their assessments, though the times were slightly less among the family physicians. Time spent using the CAM algorithm has been found to be similarly brief in its different language versions [23].

The main finding of the study is that of very strong sensitivity and specificity of the CAM algorithm for delirium when compared against a gold standard of psychiatrist diagnosis of delirium per the DSM. The study found a sensitivity of 91.9% and a specificity of 100%. As is the case for any diagnostic test, the positive and negative predictive values (PPV and NPV, respectively) of the test are a function of the prevalence of the disease. In this study, the point prevalence at the time of administering the CAM algorithm was 56.1%. At this prevalence rate, the PPV was 100%, and the NPV was 90.6%. The point prevalence of delirium, of course, is likely to vary depending on the clinical setting (e.g. inpatient versus outpatient, geriatric versus adults with some geriatric cases, surgical versus medical versus dementia populations, etc.) and may well vary over the course of hospitalization (e.g. as patient's recover, or develop hospital based infections, etc.). An example of the impact on PPV and NPV of different delirium point prevalence rates is as follows. If the prevalence of delirium drops to 10%, the sensitivity and specificity data from the current study predict that the PPV for the CAM Algorithm would remain constant (at 100%), and the NPV would increase (to almost 99%). Indeed, the PPV remains constant regardless of the prevalence rate, and the NPV would drop only in settings where the prevalence rate increases over a prevalence rate of 56.1%. These considerations argue that the CAM Algorithm is a very powerful diagnostic tool for the detection of delirium, and for screening those who likely do not have delirium, in elderly Thai inpatient populations.

## Conclusions

There were no differences found between the family physicians and psychiatrists in terms of diagnosing delirium in elderly patients. The Thai version of the CAM algorithm demonstrated a high level of accuracy when diagnosing delirium in elderly Thai patients who had been hospitalized in a non-ICU; revealing 91.9% sensitivity and 100.0% specificity, as compared to the interviews by psychiatrists, who used DSM-IV TR criteria to identify delirium. The CAM algorithm is short and easy to administer. However, training is required before using the tool, and a more fully cognitive assessment should be added to prevent false negatives, especially for patients who have dementia. Further study is encouraged regarding the use of the CAM algorithm by other medical professionals, especially bedside nurses.

## Competing interests

The authors declare that they have no competing interests.

## Authors' contributions

NW conceived and designed the study, and translated the CAM and its manual into Thai. All except PB collected the data, while PB was responsible for the statistical design of the study and for carrying out the statistical analysis. NW assisted with analyzing the data and wrote the paper. All authors assisted with manuscript writing and read the final manuscript prior to submission.

## Pre-publication history

The pre-publication history for this paper can be accessed here:

http://www.biomedcentral.com/1471-2296/12/65/prepub
